# The Melody® valve and Ensemble® delivery system for transcatheter pulmonary valve replacement

**DOI:** 10.1111/nyas.12194

**Published:** 2013-07-08

**Authors:** Doff B McElhinney, Jill T Hennesen

**Affiliations:** 1 Departments of Pediatrics, Medicine, and Cardiothoracic Surgery, NYU Langone Medical CenterNew York, New York; 2 Medtronic Inc., Cardiac and Vascular Group, Mounds ViewMinnesota

**Keywords:** Melody TPV, pulmonary valve, PPVI, failed RV to PA conduit, transcatheter pulmonary valve

## Abstract

The Melody® transcatheter pulmonary valve (TPV) is a percutaneous valve system designed for the treatment of obstruction and/or regurgitation of prosthetic conduits placed between the right ventricle and pulmonary arteries in patients with congenital heart disease. In 2000, Melody TPV became the first transcatheter valve implanted in a human; in 2006 it became the first transcatheter valve commercially available anywhere in the world; and in 2010 it was launched as the first commercially available transcatheter valve in the United States. In this review, we present the clinical background against which the Melody valve was developed and implemented, introduce the rationale for and challenges of transcatheter valve technology for this population, outline the history and technical details of its development and use, and summarize currently available data concerning the performance of the device.

## The clinical background

Approximately 8 of every 1000 babies are born with congenital heart disease, 20% of whom have deformities that involve the right ventricular outflow tract (RVOT) and pulmonary valve, which channel the deoxygenated blood returning from the body through the right ventricular (RV) pumping chamber to the pulmonary arteries. Children with complex congenital heart disease involving the RVOT, including defects such as tetralogy of Fallot, truncus arteriosus, and some forms of transposition of the great arteries, typically undergo surgical repair in the first days or months of life. As part of this repair, the RVOT is reconstructed, usually by patching or augmenting the outflow tract or inserting some form of prosthetic conduit or valve to connect the RV with the pulmonary arteries. Although these procedures accomplish the initial anatomic goals of separating the chambers of the heart and connecting the heart to the great arteries in a normal configuration, it is generally not the final solution for these patients. All of the prosthetic conduits and valves used for RVOT reconstruction, whether in children or adults, are composed of either synthetic material or nonviable homograft or xenograft tissue. Thus, they cannot grow with the patient, which is a major limitation when implanted in small children, and they are subject to progressive degeneration and dysfunction. The same problem applies to patients who receive an RVOT conduit as part of the Ross operation for aortic valve disease, a procedure in which the native pulmonary valve is relocated in the aortic position and a prosthetic conduit is used to reestablish a connection from the RV to the pulmonary arteries.

Depending on the method of reconstructing the RVOT, the pathway may become obstructed, which introduces a pressure load on the RV, and/or there may be progressively severe pulmonary regurgitation (PR), which results in an RV volume load. These lesions and the extra work they impose on the RV can be variably well tolerated over time, and often become fairly advanced before the onset of symptoms, but regardless of the clinical manifestations and pathophysiologic timeline, they are detrimental to the RV, and ultimately to the patient.

Until recently, comprehensive treatment to relieve RVOT dysfunction almost always required open-heart surgery and implantation of a prosthetic pulmonary valve or valved conduit. Accordingly, one of the ongoing issues in such patients is when to intervene. Intervening earlier in the evolving process of obstruction or PR will reduce the duration and severity of the abnormal RV load, but will entail the short-term risks associated with surgery and will start the clock ticking again—that is, once a pulmonary valve is implanted or a conduit replaced, the replacement valve generally wears out, such that there is recurrence of RVOT obstruction and/or PR over time. Eventually, the outflow tract dysfunction will become more advanced and it will be necessary to confront the dilemma of whether to wait or intervene again, weighing the risks, costs, and benefits of the two options. If a patient waits too long before relieving the RVOT dysfunction, there may be significant and irreversible damage to the RV that can have an important impact on long-term prognosis. On the other hand, repeatedly intervening early in the process, in an effort to minimize the cumulative impact of RV pressure and/or volume overload, will subject the patient to many open-heart operations, and the attendant risks and morbidities over the course of a lifetime. Clearly, the management of the RVOT in such patients is a lifelong issue. However, because of a limited understanding of how the various risks and benefits balance against one another, variability among patients, and the lack of truly long-term data (i.e., the first open-heart surgery was performed less than 60 years ago, substantially shorter than the life expectancy of a newborn in 2012), it is difficult to know how best to proceed at any given point in a specific patient, not to mention how to develop strategies that take into account the long-term perspective.

## The promise and challenges of percutaneous therapy for RVOT dysfunction

Against this clinical background, it seems obvious that a safe and effective nonsurgical approach to managing RVOT dysfunction would be highly desirable. Transcatheter implantation of a prosthetic heart valve providing some or all of the benefits of surgical valve replacement, without the risks and morbidities associated with a redo median sternotomy and open-heart surgery, has the potential to shift the risk–benefit calculation substantially in many patients. This is particularly true for transcatheter replacement of the pulmonary valve, insofar as patients with congenital heart disease involving the RVOT may require multiple open-heart surgeries over a lifetime.

However, transcatheter therapy for postoperative RVOT dysfunction offers a number of functional and technological challenges. Notably, there is considerable anatomic and physiologic variability among patients in this population. When Professor Philipp Bonhoeffer first set out to develop a transcatheter pulmonary valve (TPV), he had in mind patients with a dysfunctional surgical RVOT conduit. Such patients typically have both RVOT obstruction and PR, and in order for a transcatheter device to provide viable therapy, it would need to be able to treat both, relieving the obstruction and providing a competent valve.

The extensive anatomic variability among dysfunctional RVOT conduits—including size, length, course, material characteristics (e.g., distensible conduits, heavily calcified conduits, synthetic polymer conduits), and mechanical environment within the chest—had several important implications for the design/development process. First, it would be necessary to develop a device that could fit within the various anatomic configurations that would be encountered and still function properly, across a range of diameters that extended to full adult size. Second, there was no way to model or understand the loading conditions in all of the potential implant environments, so the optimal methods and applicability of preclinical testing were uncertain.

## The Melody transcatheter pulmonary valve system: technology and development

### Melody valve

In the late 1990s, Philipp Bonhoeffer, then in Paris, developed his first prototype for a stent-mounted biological valve intended for implant in the pulmonary position. Eventually, he refined this design by sewing a valved segment of bovine jugular vein within a modified version of a balloon expandable platinum–iridium stent that was commercially available in Europe at the time (CP Stent, NuMED Inc., Hopkinton, NY), producing a balloon expandable stent-mounted valve for TPV replacement. In February 2001, Medtronic, Inc. acquired the Contegra Pulmonary Valved Conduit (PVC) from VenPro Corp., the same company that Bonhoeffer had been working with to prototype his valve. Contegra PVC is a surgical conduit comprising a valved segment of bovine jugular vein that seemed to be the perfect valve for a percutaneous application when modified slightly. Medtronic further developed the TPV concept and conducted the necessary testing to complete the design/development process and clinical studies to obtain regulatory approvals to commercialize this technology. The valve was given the name Melody transcatheter pulmonary valve.

The Melody TPV brings together two existing device technologies: a balloon expandable stent and a valved bovine jugular venous conduit. There were important design and development considerations for each of these components separately, as well as for the integrated TPV.

Development of a valve that would perform well and be durable across a range of implanted sizes and geometries, extending to full adult size, was a critical challenge. Thus, design requirements for the tissue component of the device included not only that transcatheter delivery be feasible, including the capacity to be mounted on a flexible metal frame and crimped to a diameter no more than 7 mm safely and without damage, but also that it function well when deployed at anywhere from 14 to 22 mm in diameter, sometimes in a noncircular or noncylindrical geometry, for an extended period of time. Given these requirements, the naturally occurring bovine jugular vein valve with its very thin leaflets and deep commissures (providing exceptional coaptation of the leaflets at various internal diameters) proved to be an outstanding option. A bovine jugular venous valve measuring 18 mm diameter in its natural state was determined to be the optimal and most flexible size for this application and the target range of deployed internal diameters. Once the tissue source and specifications were specified, finding herds that could support the demand was another important challenge. Very large bulls (>400 kg) would be required to harvest suitable size jugular veins, and tissue from bulls of this size is often low yield due to an increased prevalence of anomalies in older animals and Medtronic's quality inspection requirements. The venous segments that are eventually incorporated into the Melody TPV are carefully selected from a much larger pool of veins as they go through the manufacturing and quality inspection process.

**Figure 1:**
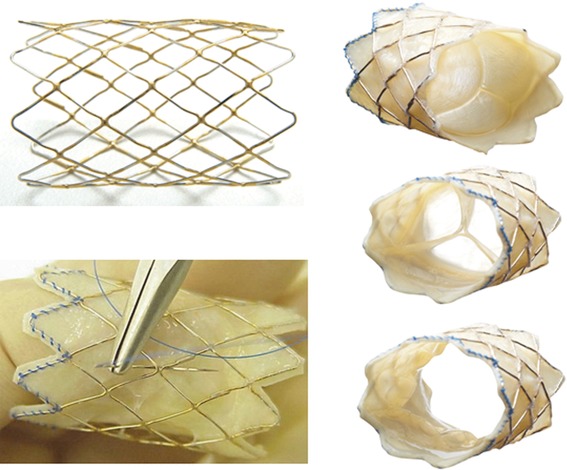
Depiction of the Melody TPV at various stages of production and in various configurations. Along the left side, the gold-brazed platinum–iridium stent is shown before attachment of the bovine jugular venous segment (top) and during the process of suturing the venous wall to the stent nodes (bottom). Along the right side, the fully assembled valve is shown in three different configurations: (top) viewed from the inflow end with the valve leaflets in a closed position; (middle) viewed from the outflow end with the leaflets in a closed position; and (bottom) viewed from the outflow end with the leaflets open.

The stent chosen for the frame by Bonhoeffer was a modified version of the closed cell CP Stent. The CP stent comprises six rows of circumferential struts, each of which is fashioned from a length of platinum–iridium wire that is welded at the ends and formed to have eight crowns (“zigs”). The individual struts are welded together at the crowns. The stent measures 34 mm in length in an unexpanded configuration and 28 mm in length when expanded to 18 mm in diameter (Fig. 1). The most important design characteristics that made this stent appropriate for use in the Melody valve platform were its relative malleability, which was felt to be important for facilitating conformation to irregular RVOT conduits, as well as easy crimping on a balloon delivery catheter, and its ability to expand well beyond the expected working diameter of the valve without shortening to a degree that would distort the valve leaflets.

After animal testing^2^ and the first TPV implantation into a human in 2000,^1^ the device underwent two important design modifications. First, the lap welds and zig-to-zig welds of the platinum–iridium stent were gold-brazed to increase their strength. Second, in 2003, the method of securing the venous segment to the frame was modified. Initially, the venous segment, which covers the full length of the frame, was sewn around the entire circumference of the frame at both the proximal and distal ends only, with no other fixation. After this version of the valve was implanted in 21 patients, it became clear that the lack of connection between the venous wall and frame in between the ends of the device was suboptimal. In some patients, a hammock effect was observed, in which the walls of the venous segment protruded into the lumen of the valve causing obstruction, due to blood passing between the outer wall of the vein segment and the frame and/or to a Venturi effect.^3^ The device was thus modified such that the vein segment was secured to the frame at every stent node with a separate interrupted suture.

The current version of the Melody TPV (Fig. 1) consists of a valved segment of bovine jugular vein that is similar to the Contegra conduit. Valve harvest, processing, and manufacturing follow a rigorous process. Fresh bovine jugular vessels are received from the slaughterhouse, inspected, tested for competency, rinsed, trimmed, and cleaned in preparation for fixation. They are fixed in glutaraldehyde, initially under pressure, then without pressure. After fixation, the veins are inspected once again for physical anomalies, tested for leakage, sized, and trimmed before valve assembly. Tissue is then sutured to the frame—using blue suture at the distal end of the valve to ensure proper orientation of the valve on the Ensemble transcatheter delivery system, which has a blue carrot tip on the end. The valve is then leak tested again, inspected microscopically for proper assembly, and cleaned and trimmed into the final product (Fig. 1). Rigorous and systematic criteria are maintained for each of these steps. The completed device is packaged and sterilized in a glutaraldehyde, isopropyl alcohol, and phosphate-buffered saline solution, in which the valve is preserved and packaged for use.

### Ensemble® transcatheter delivery system

**Figure 2:**
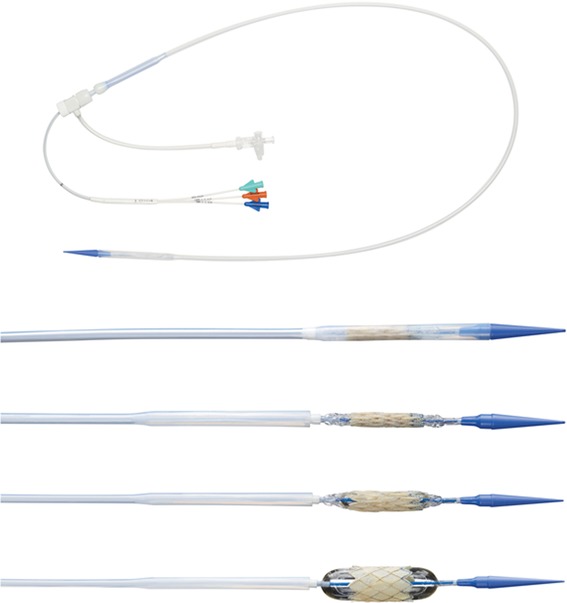
Depiction of the Ensemble transcatheter delivery system. On the top, the entire delivery system is shown. Below that, from top to bottom, the delivery system is shown with the sheath covering the valve, the sheath withdrawn so that the valve is exposed, the inner balloon of the balloon-in-balloon (BiB) inflated, and the outer balloon of the BiB fully inflated.

In conjunction with the Melody TPV, a companion delivery system was developed to facilitate introduction, delivery, and deployment of the valve (Fig. 2). The valve was much thicker than a bare metal stent, and would require a large sheath to introduce into the venous system and a mechanism to protect the valve as it was advanced to the RVOT. The Ensemble delivery system consists of an integrated balloon, long-sheath, and introducer, such that it can be introduced through the skin, delivered over a guidewire to the RVOT, and deployed without any additional vascular sheath or catheters. A balloon-in-balloon angioplasty balloon (BiB, NuMED Inc., Hopkinton, NY) was modified and mounted within a Teflon sheath that is 22 Fr in outer dimension at its distal portion, where it covers the valve, and 16 Fr along most of its length. Because the body of the sheath is smaller caliber than the portion that houses the valve, there is a tapered component that slides along the outer aspect of the sheath to provide hemostasis at the access site. The system comprises two concentric balloons: an outer balloon and an inner balloon, which is half the diameter and shorter than the outer balloon. The delivery system is manufactured with outer balloon diameters of 18, 20, and 22 mm. The tip of the balloon catheter is equipped with a blue carrot, which tapers distally to act as an introducer, and has a proximal end that engages within the distal end of the sheath to create a smooth contour. Other than the different balloon sizes, 18, 20, and 22 mm, the delivery systems are identical. The full working length of the delivery system is 100 cm.

**Figure 3:**
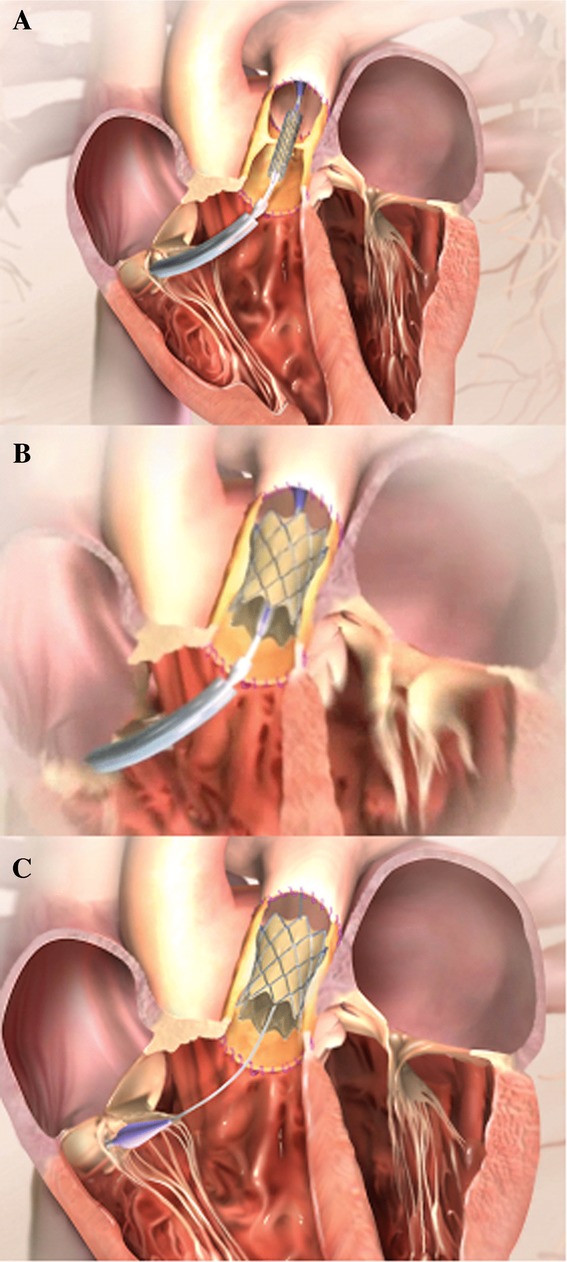
Schematic rendering of Melody® valve delivery and deployment. (A) The Ensemble delivery system has been passed through the tricuspid valve and across the right ventricular outflow tract (RVOT) conduit. The sheath has been pulled back with the valve still crimped on the balloon. (B) The Melody valve expanded by inflating the inner and outer balloons. (C) The delivery system has been withdrawn back into the right atrium, and the guidewire remains in place across the deployed Melody valve.

The Melody valve is crimped by hand onto the balloon of the appropriate sized Ensemble delivery system, after which the covering portion of the sheath is advanced over the balloon-mounted TPV (Figs. 2 and 3). When the valve is covered, it is protected and stable. The delivery system is introduced directly into the access vein over a stiff guidewire, and advanced to the RVOT. Once it is in proper position, the sheath is drawn back to expose the valve. The inner and outer balloons of the balloon-in-balloon system are inflated in succession to deploy the valve. After the valve is implanted, the balloons are deflated and the entire delivery system is removed over the guidewire (Fig. 3).

## The clinical experience with TPV replacement using the Melody valve

**Figure 4:**
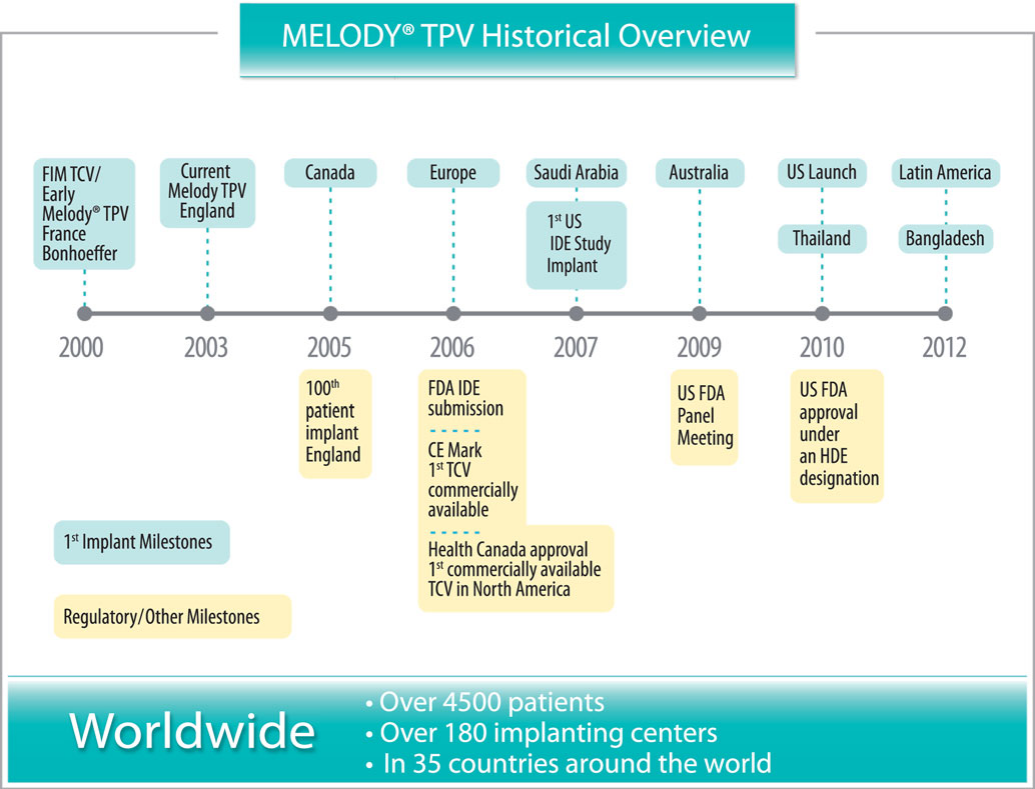
History of Melody transcatheter pulmonary valve (TPV).

A timeline of milestones in the history of the Melody valve is presented in Figure 4. As noted, TPV replacement was first reported by Philipp Bonhoeffer in 2000.^1^ He performed 10 cases in Paris, after which he moved to Great Ormond Street Hospital in London, where he continued his program. After 21 implants, the valve was redesigned to prevent the hammock effect discussed earlier.^3^ In May 2005, a database of Bonhoeffer's first 89 implants was closed for regulatory submission for CE Mark approval in Europe and a proposed investigational device exemption (IDE) trial protocol in the United States. In September of that year, the 100th Melody valve was implanted. Then, in 2006, the Melody valve received CE Mark approval in Europe and Health Canada approval, making it the first commercially available transcatheter valve in the world.

In 2007, a prospective nonrandomized IDE trial began at three centers in the United States, with the first Melody valve implant in the United States performed in January of that year.^4^ After the addition of two more investigative sites and several protocol amendments that increased the total number of implants allowed, trial enrollment was completed and the 150th implant was performed in January 2010. That same month, the Melody valve was officially approved in the United States under a Humanitarian Device Exemption (HDE), with the stipulations that patients enrolled in the IDE trial were followed for the intended five-year period and that additional data collection continues in a postapproval study cohort. In the United States, the instructions for use under the HDE approval specify that the Melody TPV is indicated for use as an adjunct to surgery in the management of pediatric and adult patients with the following clinical conditions:

- existence of a full (circumferential) RVOT conduit that was equal to or greater than 16 mm in diameter when originally implanted; and
- dysfunctional RVOT conduit with a clinical indication for intervention and either:
  - regurgitation: ≥ moderate regurgitation, or;
  - stenosis: mean RVOT gradient ≥35 mmHg.

The first commercially approved Melody valve implant in the United States was performed in February 2010. By the end of 2012, 85 centers in the United States had received training to implant the Melody valve, and over 4500 valves had been implanted in 35 countries worldwide.

## Device performance

**Figure 5:**
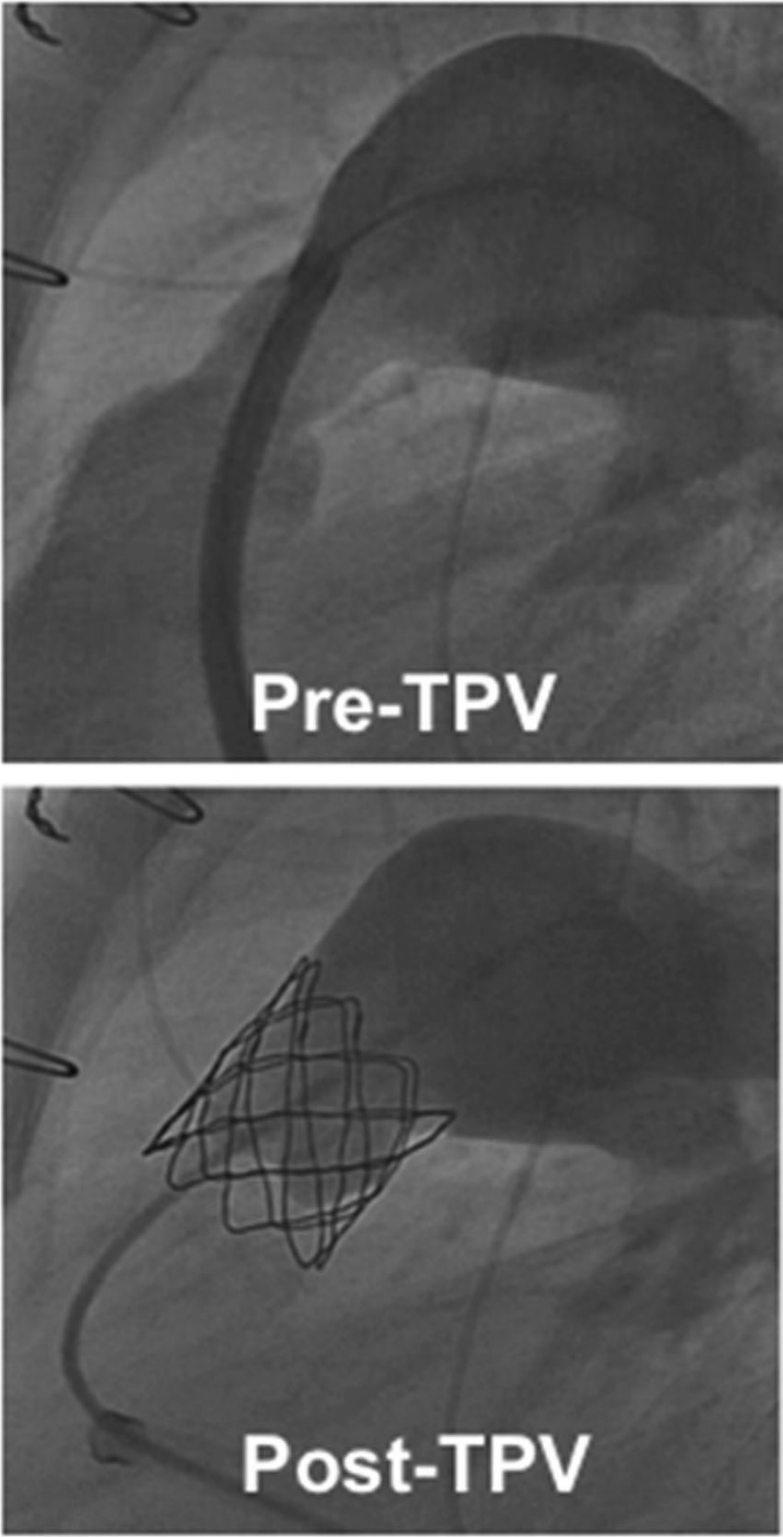
Angiograms in the lateral projection before (top) and after (bottom) Melody transcatheter pulmonary valve (TPV) implant in a right ventricular outflow tract (RVOT) conduit that was both obstructed and regurgitant.

Reports from single and multicenter studies in Europe, Canada, and the United States have consistently found that in properly selected patients, the Melody valve can be implanted in the intended location in the RVOT with few serious procedural complications.^3^–^15^ These studies have also clearly and consistently demonstrated that the Melody valve provides effective acute relief of both obstruction and PR (Fig. 5), and leads to improvement in biventricular efficiency, functional status, and symptoms, with minimal morbidity and a high level of safety.^3^–^20^ Given the range of physiologic abnormalities among treated patients, ranging from pure RVOT obstruction to pure PR, with mixed obstruction and PR the most common, the specific functional and physiologic outcomes of TPV replacement have been found to vary.^4^–^20^ For example, in patients with PR, providing a competent pulmonary valve by implanting a Melody TPV reduces RV volumes and improves some measures of left ventricular diastolic function. In patients with significant RVOT obstruction, the Melody valve implant improves exercise cardiopulmonary function, ventricular systolic function, and early left ventricular (LV) diastolic filling.

In early experience, both in Europe and in the U.S. IDE trial, fracture of the Melody valve frame was the most frequent event noted.^7^–^21^ In some cases, there was fracture of one or more stent struts with no apparent functional compromise, but in others, there was clear loss of stent integrity and recurrent RVOT obstruction related to the stent fracture. However, standard practice has evolved to include the practice of prestenting, namely, placement of one or more nonvalved balloon expandable stents at the site of obstruction, and then implanting the valve into the conduit.^7^–^22^ On the basis of recent studies, prestenting has become a common practice and greatly mitigates the risk of fracture^a^.^7^–^21^ Because stent fracture is a time-dependent phenomenon, there are no long-term data regarding the impact of prestenting, which warrants continued investigation.

Functional failure of the Melody valve has been uncommon and has occurred almost exclusively as recurrent RVOT obstruction in the context of a fractured stent. Accordingly, almost all re-interventions following implant of the valve have been to relieve the resultant obstruction.^23^ As with all bioprosthetic valves, endocarditis has been observed, and in some instances has resulted in explant of the valve.^24^ Significant regurgitation of the Melody valve has been very rare, and in this respect, the performance of Melody TPV has exceeded expectations.

## The future

The Melody valve was the first transcatheter valve implanted in a human, and is likely to alter fundamentally the management of RVOT dysfunction in patients with complex congenital heart disease. It is truly a revolutionary medical device, although there is much that remains to be learned in order to understand its performance, as well as its potential drawbacks, and how to most effectively incorporate it into clinical practice. Moving forward, there are a number of important ongoing initiatives in the Medtronic Structural Heart Pulmonic program, which are intended to improve patient outcomes, expand the reach of TPV therapy, and continue to reduce the number of open-heart surgeries required in this patient population.

A major initiative in Medtronic's Pulmonic program is to expand TPV therapy to a broader population of patients with RVOT dysfunction who are not currently candidates for the Melody valve. At present, the Melody TPV is approved only for patients with dysfunction of an RVOT conduit (approximately 15% of patients with congenital heart disease in whom pulmonary valve replacement is indicated). Extensive research and development is being conducted to design a suitable transcatheter device for patients who have a dysfunctional native or patch-augmented RVOT, typically with significant PR rather than obstruction, that is too large for the current Melody valve.^25^–^26^ There is tremendous variability in the dynamic RVOT anatomy among patients in whom a patch-augmentation repair was performed.^25^–^26^ Accordingly, one of the major challenges in this is constructing a device or devices that can effectively serve this incredibly diverse population. It is anticipated that a transcatheter valve designed for the large RVOTs will serve approximately three to four times as many patients as current Melody TPV therapy. The first in-human implant of a large RVOT TPV device developed by Medtronic was reported by Bonhoeffer's group in 2010.^27^ Further research and development of designs based on this model are underway.

## Other advances related to the launch of Melody TPV therapy

An essential correlate to the introduction of any new technology, particularly one with unique and unprecedented technical requirements, is ensuring appropriate training in its use. As part of the broader transcatheter valve program at Medtronic, state-of-the-art physician education programs have been developed to ensure patient safety and optimal clinical results. This training includes the use of virtual reality implant simulators for transcatheter valve implantation, as well as peer-to-peer physician training and proctored cases.

In addition, the process of developing the Melody TPV has provided an important foundation both as a product platform and for implementing clinical and regulatory pathways for other Medtronic transcatheter products to follow. Importantly, the processes and experience Medtronic gained with the Melody TPV has and will help set the stage for the launch of future Medtronic transcatheter products for the replacement and/or repair of both the aortic and other heart valves, which will help meet a substantially larger clinical need than that of the congenital heart patients served by the Melody TPV.

*Note*: The HDE was authorized by federal law (USA) for use in pediatric and adult patients with a regurgitant or stenotic right ventricular outflow tract (RVOT) conduit (≥16 mm in diameter when originally implanted). The effectiveness of this device for this use has not been demonstrated.

## Conflicts of interest

J.T.H. is an employee of Medtronic Inc. D.B.M. is a paid proctor and consultant for Medtronic Inc. and was an investigator in the Medtronic Melody TPV U.S. IDE study.

## Footnotes

a Note: although prestenting has become a current practice, it is currently considered an off-label use, as no devices are approved in the United States for prestenting.
